# The Alongside Digital Wellness Program for Youth: Longitudinal Pre-Post Outcomes Study

**DOI:** 10.2196/73180

**Published:** 2025-10-08

**Authors:** Katherine Cohen, Andy Rapoport, Elsa Friis, Shannon Hill, Sergey Feldman, Jessica Schleider

**Affiliations:** 1 Department of Medical Social Sciences Feinberg School of Medicine Northwestern University Chicago, IL United States; 2 Alongside Care Seattle, WA United States

**Keywords:** digital mental health, schools, youth, Alongside, LGBTQ

## Abstract

**Background:**

Youth are increasingly experiencing psychological distress. Schools are ideal settings for disseminating mental health support, but they are often insufficiently resourced to do so. Digital mental health tools represent a unique avenue to address this gap. The Alongside digital program is one such tool, intended as a universal prevention and early intervention. The platform includes social-emotional learning and self-help wellness features as well as an artificial intelligence–powered chatbot designed to build coping skills.

**Objective:**

This evaluation aimed to examine the near-term impact of Alongside app use on students’ self-reported mental health outcomes.

**Methods:**

We conducted a nonrandomized pilot pragmatic evaluation leveraging anonymized user data. All data came from current Alongside users attending public middle and high schools in Texas and New Mexico, between 10 and 18 years old. Schools were actively engaged in partnership with Alongside and approved all procedures. Users were asked to complete mental health questionnaires upon app registration and at 1 and 3 months post registration. We conducted preregistered analyses as well as exploratory analyses to determine how symptoms changed over time and what factors (eg, demographic and app use) predicted changes in distress.

**Results:**

Analyses revealed statistically significant within-person decreases in overall distress (Young Person’s CORE; primary outcome) from baseline to 1 month with a small effect size (*t*_42_=2.21, *P*=.03, *r*=0.34); however, there was no evidence that scores significantly decreased from baseline to 3 months (*W*=1821, *n*=85, *P*=.16). We found that at 3 months, identifying as part of the lesbian, gay, bisexual, transgender, queer, and questioning community predicted greater decreases in distress; otherwise, no demographic factors were significant predictors. In a nonregistered exploratory analysis of a subsample of users who reported elevated distress at baseline, decreases in distress were seen at both 1 month (*W*=128, *n*=20, *P*=.02, *r*=0.52) and 3 months (*W*=682, *n*=42, *P*=.004, *r*=0.45).

**Conclusions:**

There may be short-term benefits associated with using the Alongside digital program. Further studies are required to determine potential preventative effects.

## Introduction

Children and adolescents are increasingly experiencing psychological distress and mental health concerns; from 2009 to 2019, the proportion of youth in the United States who reported persistent feelings of sadness or hopelessness rose from 26% to 37% [1]. The COVID-19 pandemic exacerbated already concerning mental health outcomes in youth, with reports suggesting that minoritized youth (eg, homeless youth, LGBTQ [lesbian, gay, bisexual, transgender, queer, and questioning] youth, and youth of Color) experienced a disproportionately negative effect of pandemic stressors [2,3]. Unfortunately, access to needed mental health care for youth is limited by both structural (eg, financial) and attitudinal barriers (eg, stigma) [4]. Further, there are increasing calls to approach the youth mental health crisis from a population health perspective by teaching positive coping skills to all youth, which may serve to prevent subclinical distress from progressing to the point of needing intervention [5,6]. To address the rising rates of distress in youth, multilevel solutions are needed that can universally promote wellness while also linking students with additional needs to supports that can be accessed with minimal barriers or delays.

One of the most common settings where youth attempt to access mental health care is schools [7], likely due to schools being a unique setting in which certain barriers to access (eg, cost and location) are comparatively minimal. Additionally, schools are an ideal setting for universal prevention and early intervention efforts, as they are one of the only settings where the majority of a population (ie, youth) are found in one place, and the already educational nature of schools is consistent with providing psychoeducation surrounding mental well-being [8,9]. Students, particularly those who are traditionally underserved, are more likely to receive mental health support and wellness resources in schools independent of structural and policy challenges they may face externally, in their homes and communities [10]. Schools are overwhelmed by mental health needs among their students, however, and are often incapable of meeting the needs of all students who seek services or could benefit from preventative efforts [11,12]. Although some support and funding were made available during the pandemic to schools, those are likely not continuing [13]. To prevent worsening of outcomes and provide support as quickly as possible, schools need cost-effective solutions that do not strain an already overstretched system, while filling gaps that existing support cannot.

Digital mental health tools and interventions may be viable options for support that can add to an array of school-based services. Digital technology is widely used by young adults [14]. Many youth already use digital tools in efforts to aid their mental health and everyday distress, with more than 80% using an internet search to find mental health information [15]. Digital resources offer the opportunity for 24/7 support, meaning they can be accessed during acute moments of distress. Further, many students may be hesitant to ask for in-person support due to concerns around stigma; alternatively, they may wish to address issues themselves, as youth often report the desire for self-autonomy [4]. Critically, digital tools cannot and should not replace other school-based mental health supports, such as services delivered by a licensed school-based provider. Rather, they can be cost-effective solutions to deliver psychoeducational and skill-based content for universal or selective prevention. Considering the multitiered systems of support model, a widely used framework for categorizing and implementing school-based mental health supports, digital tools may be best used as “Tier 1” supports for all students, or “Tier 2” supports for students identified as at-risk for developing mental health disorders. Such tools need to be non-stigmatizing and promote student enrollment and participation in a way that is developmentally appropriate, flexible to reach them at the times and in the ways in which they need support, in a student’s native language, and fundamentally facilitate their sense of autonomy in promoting well-being.

The Alongside digital program was designed by experts in digital mental health, ed-tech, and education to leverage the benefits of digital mental health solutions and provide schools with a scientifically grounded and cost-effective method for bolstering student well-being without overburdening staff. Alongside aims to provide personalized social-emotional learning and self-help wellness support to students in 4th through 12th grades across the country and identify students in need of higher levels of support. Alongside includes features such as psychoeducational and mindfulness resources, a 24/7 chatbot that allows students to engage in personalized skill building, and student-initiated onramps to seek additional support. Our objective during this pilot pragmatic evaluation was to leverage anonymized user data to examine the near-term impact of the Alongside app use on student self-reported clinical and well-being outcomes. We tested the following preregistered hypotheses:

- Hypothesis 1: we hypothesized that completion of at least one chat within the app (outside an initial onboarding session) would be associated with significant decreases in overall distress, depression, and anxiety symptoms, as well as hopelessness and loneliness, and significant increases in mental health treatment expectancies at 1 month and 3 months post app registration. Overall distress was our primary outcome, while other measures were preregistered as secondary outcomes.
- Hypothesis 2: we hypothesized that users would show similar decreases in overall distress across sociodemographic groups.
- Hypothesis 3: we hypothesized that greater engagement in the app (ie, multiple chat sessions) would predict larger improvements in overall distress among users.

We also tested hypotheses that we did not preregister as exploratory: (1) we tested each hypothesis separately for users with elevated symptoms at baseline; (2) we tested whether users who showed clinically significant decreases in distress differed in their app use from users who did not show clinically significant decreases in distress; and (3) we tested whether LGBTQ users differed in their app use or baseline distress from non-LGBTQ users. We were interested in the potential differences between LGBTQ users and non-LGBTQ users for two reasons: (1) LGBTQ youth are more likely to experience mental health concerns such as depression and therefore may differentially benefit from receiving mental health support [16]; (2) LGBTQ youth often face barriers receiving traditional care (eg, reluctance to disclose identity to parents and difficulty finding a culturally competent provider) and may be more likely to benefit from digital, self-help resources [17]. Exploratory analyses are described in more detail subsequently in the methods section.

## Methods

### Sample

This paper reports results from a nonrandomized, open, pragmatic evaluation using deidentified data. All participants were current Alongside users enrolled in public middle and high schools in Texas and New Mexico. Users were between the ages of 10 and 18 years. Upon app registration, users completed a set of questionnaires that asked about their mental health symptoms and help-seeking expectancies (additional details provided below). Approximately 1 month and 3 months after app registration, users were asked to complete the questionnaires again via an in-app notification. All questionnaires were available in Spanish and English. Participants were given a month to complete the questionnaires. Teachers and counselors at schools were allowed to answer student questions about the questionnaires or define words. There were no upper limits on sample size. Additionally, Alongside collected app use data (eg, which features of the app users engaged in, and how frequently) throughout the 3-month period. All data (ie, questionnaire data and usage data) were collected internally by Alongside as part of their ongoing efforts to evaluate and improve their program. The deidentified, user-level data was provided to researchers at Northwestern University for users who met the following criteria: (1) completed at least one chat within the app outside of the initial rollout session (ie, onboarding session) and (2) consented to allow their data to be used for research purposes. As all data were part of a completely anonymous evaluation, this analysis was deemed as nonhuman subjects research in consultation with the institutional review board at Northwestern University. This paper adheres to reporting standards for secondary data analyses [18].

### Alongside

#### Overview

The Alongside app contains multiple features designed to provide personalized support for mental health. An initial prototype was developed in June 2022 based on over 50 in-depth interviews with students and school stakeholders. The first version of Alongside was piloted across the country during the 2022-2023 school year. Data from pilots and a 3-month collaborative codevelopment process with teens refined Alongside’s theory of change and features.

At the time of this evaluation, Alongside provided personalized skill building through matching a mix of rule-based and highly regulated generative artificial intelligence (AI) chats grounded in Alongside’s skill-building framework called EMPOWER: Engage (provide empathy and validation), Motivate (help the user understand how a skill helps them), Practical examples (learn through practical examples), Operationalize (apply the skill to their own life), Work on it (put the skill into action in real life), Evaluate (follow-up on how it goes), and Reinforce (celebrate progress and effort, not just the outcome). The EMPOWER framework was developed using concepts from motivational interviewing, common factors theory, and self-determination theory and was informed by the qualitative review and coding of over 5000 chats. Skills encompassed multiple theoretical approaches, including 3rd wave CBTs, including DBT, ACT, Mindfulness-based approaches, narrative therapy, social skills training, positive psychology, and solution-focused therapy. While the EMPOWER framework is scientifically grounded, development is ongoing, and the effectiveness of the framework has yet to be rigorously evaluated. Content covered within chats includes mental health coping, academic success, relationship skills, self-awareness, social awareness, responsible decision-making, and self-management. Several of these skill domains map onto social-emotional learning domains identified by CASEL. Chats are meant to be stand-alone supports, such that any one chat session is intended as a self-contained coping resource. Chats could be conceptualized as single-session interventions, which are theoretically grounded in the idea that meaningful insights can occur at any moment and change is possible within one encounter [19]. Therefore, chats are not only available once but are available one at a time.

**Figure 1:**
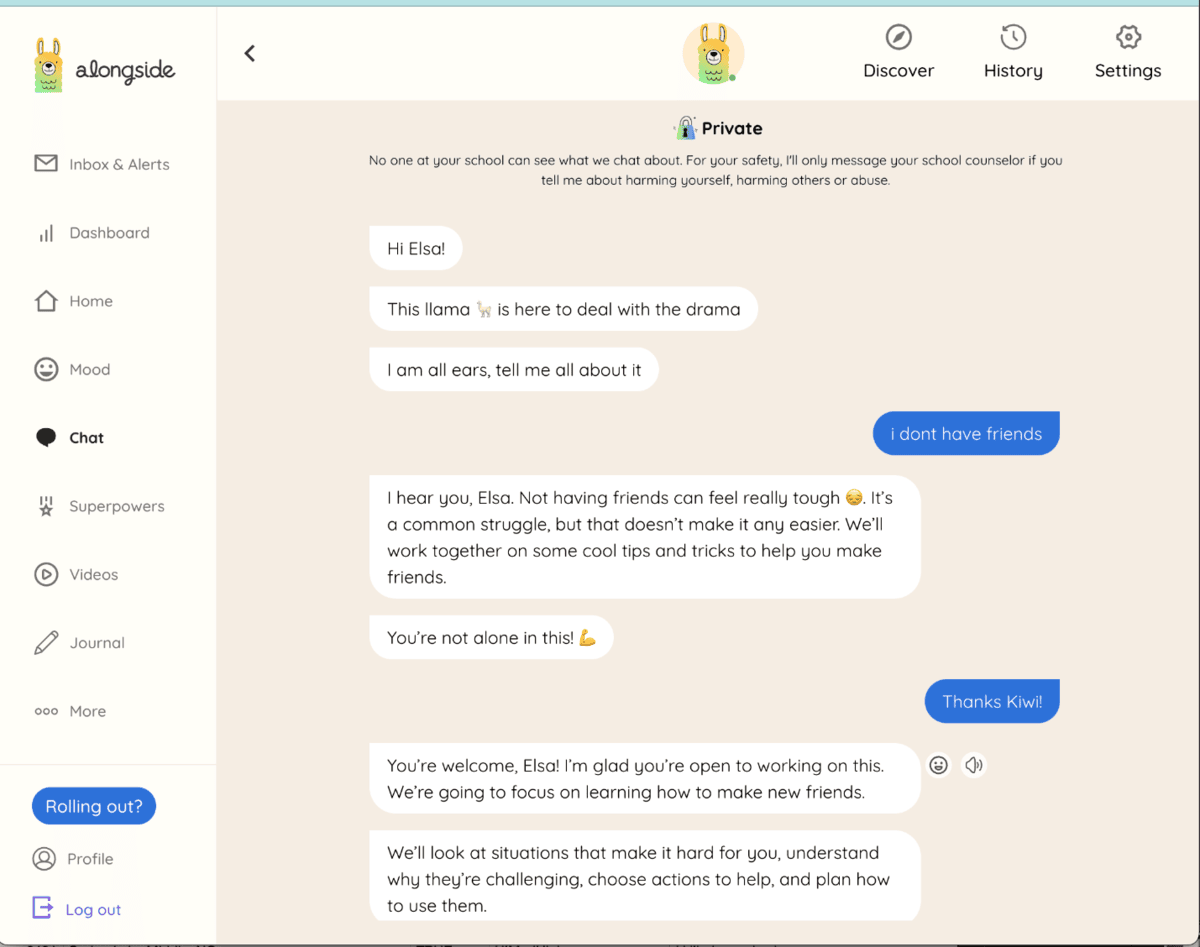
Example chat within the Alongside platform.

In the version of the app that was implemented when the data from this evaluation was collected (between January and June 2024), the chatbot included over 120 rule-based and generative AI chat “modules” that aligned with over 50 common challenges students reported in pilot data (ie, building healthy friendships, boosting self-esteem, reducing anxiety). A screenshot with an example chat is presented in Figure 1. Alongside is a member of the EDSAFE AI Industry Council and takes a multifactor approach to ensuring the safe use of generative AI. At the time of this evaluation, generative AI was used to provide validating statements to students, such as “I understand it must be hard to feel so alone” to add personalization. Users could engage with chats in Alongside in over 30 languages. Students are aware that all engagement on Alongside is confidential unless they express a risk of harm to themselves, someone else, or report abuse or harassment. Below, we include additional information on Alongside’s procedures in these instances. Outside of severe issues, students have autonomy to send a message or share a summary of their chat with their school counselor. Other activities available in the app include psychoeducational videos (eg, “The Power of Speaking Up: How Asking for Help Changed My Life”), a goal-setting tool, and a journaling tool. A mood-tracking feature was launched in February 2024. Additional user interface/user experience and content changes occurred during the evaluation period, including the creation of additional chat modules.

#### Risk Procedures

Alongside uses a proprietary machine learning safety monitoring system to screen all student free text inputs and identify any mention on self-harm, suicidal ideation, homicidal ideation, abuse or harassment. If potential risk is detected, the bot asks, “I am concerned you are at risk of serious harm, did I get that right?” The student either selects yes or no. If the student selects yes, they are asked, “Can you confirm what is happening?” Options include “thinking of hurting myself, “thinking of hurting someone else,” “worried about someone hurting me,” or “someone else needs help.” If students say no, one of Alongside’s clinical team members reviews the chat within 24 hours to ensure this was in fact a false trigger. If a student confirms a severe issue, the bot administers a self-reported severity measure such as the Columbia Suicide Severity Rating Scale, directs the student to call a 24/7 hotline such as 988, and walks the student through creating a safety plan based on their specific situation. Concurrently, an automated email and text message is generated to notify school staff of the concerning message and information gathered in the risk screening process. In addition, schools have access to a dashboard with the above information, allowing schools to easily track or refer back to important safety information. In addition to these standard procedures, if a student responded positively to “I have thought about harming myself” on the Young Person’s CORE (YP-CORE) (as described below), the Alongside program automatically matched their user ID to their name/email and generated a real-time notification to the school reporting the student name, email, and response on the survey so the counseling team could follow up and provide support. School student wellness staff have access to a dashboard including deidentified data on app use, common challenges or topics students are discussing, as well as information on students with severe issues or who choose to reach out.

All data on Alongside is protected via industry-standard SSL-encryption. Deidentified data was transferred to Northwestern via a secure file transfer. A full explanation of Alongside’s data security procedures can be found on the Alongside website. Northwestern stored the data in a secure, encrypted server.

### Recruitment

During the fall of 2024, Alongside approached 4 schools that had purchased Alongside, who represented the typical school that partners with Alongside to share deidentified data for research purposes prior to rolling out the platform to students. One additional school was recruited from a district that had previously implemented Alongside in multiple schools and was open to providing Alongside to a school that had not previously used it in order to ensure there was representation from an Urban district. All 5 middle and high schools agreed to partner with Alongside to provide the program to their students and share deidentified data for research purposes. Table 1 provides general information about each school as reported by the National Center for Education Statistics [20].

**Table 1:** Characteristics of the 5 middle and high schools that agreed to provide Alongside to students and share deidentified data.

School	Location	Type	Grades	Size	Urban/Rural	Percentage free or reduced lunch	Diversity^a^
School 1	New Mexico	Public	9th-12th	621	Remote town	100%	76% BIPOC^b^
School 2	Texas	Public	9th-12th	1157	Distant town	81%	90% BIPOC
School 3	Texas	Public	6th-8th	750	Suburban	86%	99% BIPOC
School 4	Texas	Public	6th-7th	561	Distant town	74%	90% BIPOC
School 5	Texas	Public	6th-8th	772	Large city	93%	94% BIPOC

^a^Percentage BIPOC is the percentage enrollment by non-White race/ethnicity, that is, the percentage enrollment of American Indian/Alaska Native, Asian, Black, Hispanic, Native Hawaiian/Pacific Islander, and 2 or more races.

^b^BIPOC: Black, Indigenous, and People of Color.

This study is a naturalistic implementation study and thus all procedures followed the typical roll-out procedures, Alongside uses across all schools. To implement Alongside, school counseling teams must complete a 45-minute orientation. Additionally, schools must determine parental consent/assent procedures prior to implementing Alongside. Each district has a protocol, largely determined by their superintendent, regarding whether parents must give active consent for their child to use Alongside, or whether parents may choose to opt out of providing consent for their child to use Alongside. All schools included in the current evaluation opted for a parental opt-out procedure for using Alongside and sharing deidentified data for research purposes. School 3 made the decision to exclude students younger than 13 years from using the app. All other schools made the decision that this process was sufficient per their school/district/state regulations. Letters were sent by the schools to all parents via their typical communication method (eg, email and Canvas) to provide information about Alongside, including a statement that deidentified data would be used for research purposes. Letters were sent in both English and Spanish. Parents were given the opportunity to opt their child out of accessing the Alongside program or opt out of deidentified data sharing at any time. No parents opted out of either.

Schools or districts that had active partnership agreements with Alongside but had not yet begun to implement the program with students by January 2024 were invited to participate in the research. All schools were recommended to roll out Alongside via a 20-minute teacher-led roll-out activity during class. Schools are provided with posters and cardboard cutouts to promote awareness of Alongside. Additionally, school counseling and wellness staff receive a monthly newsletter with topical suggestions. For example, in February, schools received a newsletter with recommended journal prompts, videos, and chat starters focusing on healthy dating relationships and teen dating violence prevention.

### Ethical Considerations

Alongside adheres to all state and school district regulations concerning the use of AI in their product, topics that they cover, and parental consent procedures. Quantitative measures used in this study aligned with school universal screening procedures which is a service Alongside provides, therefore additional permission was sought to share deidentified data for research purposes. Parents were able to opt their children out of the Alongside platform for sharing deidentified data at any time. In addition, students were asked to provide consent to share their deidentified data with Northwestern University. Across all schools, 55.4% (1356/2448) of students consented to share deidentified data. Compensation was not provided to participants. All data was deidentified prior to being shared with the external Northwestern research team. As all data were part of a completely anonymous evaluation, this analysis was deemed as nonhuman subjects' research in consultation with the institutional review board at Northwestern University (STU00220768). There is no identification of participants in the text of this manuscript, figures/tables, images, or any related materials.

### Measures

#### Demographics

Upon app registration, users were asked to report their age, sex (female, male, and prefer not to say), race (American Indian or Alaska Native, Asian, Black or African American, Hispanic or Latino, Native Hawaiian or Other Pacific Islander, or White) and whether they identified as someone who was part of the LGBTQ community (yes or no).

#### The YP-CORE

The YP-CORE was our preregistered primary outcome. This 10-item scale asks participants to indicate how often they experience symptoms of distress (eg, “I’ve felt edgy or nervous”) on a 5-point Likert scale ranging from 0 (not at all) to 4 (most or all of the time). Scores are summed and range from 0 to 40, with higher scores indicating greater levels of distress [21]. Among the users who completed the baseline and 1 month questionnaires, internal consistency (α) was .80 at baseline and .80 at 1 month. Among the users who completed the baseline and 3-month questionnaires, internal consistency (α) was .79 at baseline and .79 at 3 months.

#### Patient Health Questionnaire-2

The Patient Health Questionnaire-2 (PHQ-2) asks participants how often they’ve experienced symptoms of depression (eg, “Little interest or pleasure in doing things”) over the last 2 weeks on a 4-point Likert scale ranging from 0 (not at all) to 3 (nearly every day). Scores are summed and range from 0 to 6, with higher scores indicating greater levels of depression [22]. Among the users who completed the baseline and 1 month questionnaires, internal consistency (α) was .69 at baseline and .72 at 1 month. Among the users who completed the baseline and 3-month questionnaires, internal consistency (α) was .69 at baseline and .74 at 3 months.

#### Generalized Anxiety Disorder Questionnaire-2

The Generalized Anxiety Disorder Questionnaire-2 (GAD-2) asks participants how often they’ve experienced symptoms of anxiety (eg, “Feeling nervous, anxious or on edge”) over the last 2 weeks on a 4-point Likert scale ranging from 0 (not at all) to 3 (nearly every day). Scores are summed and range from 0 to 6, with higher scores indicating greater levels of anxiety [23]. Among the users who completed the baseline and 1 month questionnaires, internal consistency (α) was .87 at baseline and .67 at 1 month. Among the users who completed the baseline and 3-month questionnaires, internal consistency (α) was .81 at baseline and .81 at 3 months.

#### Beck Hopelessness Scale-4

The Beck Hopelessness Scale-4 (BHS-4) asks participants to indicate their current level of hopelessness through rating their agreement with statements (eg, “My future seems dark to me”) on a 4-point Likert scale from 0 (absolutely disagree) to 3 (absolutely agree). Scores are summed and range from 0 to 12, with higher scores indicating greater levels of hopelessness [24]. Among the users who completed the baseline and 1 month questionnaires, internal consistency (α) was .83 at baseline and .78 at 1 month. Among the users who completed the baseline and 3-month questionnaires, internal consistency (α) was .84 at baseline and .86 at 3 months.

#### The University of California, Los Angeles Loneliness Scale-3

The UCLA (University of California, Los Angeles) Loneliness Scale-3 (ULS-3) asks participants to indicate how often statements reflecting loneliness (eg, “I feel left out”) are descriptive of them on a 4-point Likert scale from 1 (never) to 4 (often). Scores are summed and range from 4 to 16, with higher scores indicating greater levels of loneliness [25]. Among the users who completed the baseline and 1 month questionnaires, internal consistency (α) was .77 at baseline and .80 at 1 month. Among the users who completed the baseline and 3-month questionnaires, internal consistency (α) was .70 at baseline and .82 at 3 months.

#### Expectancies

Participants’ expectancies regarding mental health treatment were assessed using one item: “On a scale from 1 (not at all helpful) to 10 (extremely helpful), how helpful do you think individual therapy would be in reducing mental health problems, if you struggle/were to struggle with them?” [26].

#### App Usage Data

Alongside collected data over the 3-month period on the following usage metrics per-user: number of app opens, number of chat sessions started, number of chat sessions completed, number of chat messages sent, number of goals created, number of journal entries, number of videos watched, number of mood log entries, and total number of activities completed.

#### Data Analysis

The RStudio Statistical Program (R Foundation for Statistical Computing)) was used to complete data analyses [27]. For each statistical test, a *P* value of <.05 was considered statistically significant. Descriptive statistics for participant characteristics (eg, sex) as well as usage metrics (eg, number of chats completed) were reported in terms of means, SDs, and percentages. Cronbach α was calculated to evaluate the internal consistency of each quantitative measure at each time point. We used list wise deletion to exclude users who did not have complete data available for outcomes of interest. We additionally conducted either paired *t* tests or Wilcoxon signed-rank tests to determine whether demographic or clinical characteristics at baseline differed between participants who completed the 1 month assessment and participants who completed the 3-month assessment.

#### Hypothesis 1

To test hypothesis 1, we conducted either paired *t* tests or Wilcoxon signed-rank tests. We first tested the assumption of normality (via the Shapiro-Wilk normality test). If the assumption was met, we conducted a paired *t* test. If the assumption was not met, we conducted a Wilcoxon signed-rank test. We additionally calculated Cohen d effect sizes (with 95% CIs). We ran one set of tests comparing outcomes from baseline to the 1-month timepoint, and another set of tests comparing outcomes from baseline to the 3-month timepoint.

#### Hypotheses 2 and 3

To test hypotheses 2 and 3, we conducted multiple linear regressions. For hypothesis 2, the outcome variable was YP-CORE scores at the follow-up time-point, and predictor variables included baseline YP-CORE scores and demographic variables (age, race/ethnicity, sex, school, and whether or not they identified as a member of the LGBTQ community). We ran one model with YP-CORE scores at the 1-month timepoint as the outcome variable and one model with YP-CORE scores at the 3-month timepoint as the outcome variable. Race/ethnicity, sex, and LGBTQ status were dummy coded, with “White/Caucasian,” “male,” and “No” as the reference groups. School was dummy coded with School 1 as the reference group. For hypothesis 3, the outcome variable was YP-CORE scores at the follow-up timepoint, and predictor variables included baseline YP-CORE scores and user engagement variables. Each user engagement variable was tested individually; therefore, 10 models were run (total number of activity sessions, total number of videos watched, total number of chat sessions, total number of journal entries, and total number of goals set; 5 models for 1-month outcomes and 5 models for 3-month outcomes).

#### Additional Analyses

We additionally tested all hypotheses on a subsample of users who had clinically elevated distress at baseline (defined as YP-CORE of ≥14.1) [28]. We conducted tests to determine whether users who saw a clinically significant decrease in distress (defined as a decrease of ≥ 7.9) [27] differed in their usage data from users who did not see a clinically significant decrease in distress. We conducted tests to determine whether LGBTQ users differed in their usage data from non-LGBTQ users.

#### Preregistration

This analysis was preregistered on Open Science Framework [29]. There were several deviations from the preregistered analyses. We preregistered that we would run longitudinal analyses including all 3 timepoints (baseline, 1 month, and 3 months). However, we did not have sufficient data to run these analyses. Instead, we analyzed outcomes from baseline to 1 month, and baseline to 3 months. Additionally, we preregistered that we would analyze whether grade predicted changes in overall distress (hypothesis 2) and whether total number of minutes spent in the app predicted changes in overall distress (hypothesis 3). However, these data were unavailable. We did not preregister but chose to run the following exploratory analyses: (1) testing hypotheses separately for users with elevated symptoms at baseline; (2) testing whether users who showed clinically significant decreases in distress differed in their app use from users who did not show clinically significant decreases in distress; and (3) testing whether LGBTQ users differed from non-LGBTQ users in their baseline YP-CORE scores or app use metrics. The decision to add this set of analyses was made in light of emerging questions regarding who the app is most effective for, and which components drove effectiveness. Particularly, the decision to test whether LGBTQ users differed in their app use from non-LGBTQ users was made due to findings from hypothesis 2 as discussed in the results. Last, we preregistered that we would exclude participants younger than 12 years old, however the sample size was smaller than we anticipated. To increase the available sample size and thereby the likelihood of detecting true effects, we elected to include participants who were at least 10 years old.

## Results

### Participant Retention

Across the 5 schools, there were 3419 students who were eligible to use Alongside. There was a total of 3861 students at schools 1-5 (Table 1); however, school 3 excluded students aged 13 years or younger from this study, which removed 442 students from eligibility. A total of 2804 students signed into Alongside. Among these, 1356 consented to sharing deidentified data for research purposes. There were students who enrolled in the study on mobile, and the survey was not available on mobile; therefore, those students did not have the chance to participate. A total of 1114 students who consented to share deidentified data completed the baseline questionnaires upon registration; 301 (27%) of these students completed at least one chat outside of the onboarding session. A total of 199 students who consented to share deidentified data completed the 1-month questionnaires; 66 (33%) of these students completed at least one chat outside of the onboarding session. A total of 300 students who consented to share deidentified data completed the 3-month questionnaires; 116 (38%) of these students completed at least one chat outside of the onboarding session.

### Participant Characteristics

A total of 66 users completed both the baseline and the 1-month questionnaires. A total of 116 users completed both the baseline and the 3-month questionnaires. A total of 26 users completed questionnaires at all 3 time points. Among users who completed both the baseline and the 1-month questionnaires, 97.01% (*n*=65) chose to complete the questionnaires in English. They ranged in age from 10 to 18 (mean 14.02, SD 2.05) years old. The majority were female (32/61, 52.46). The most commonly self-identified race/ethnicity was Hispanic or Latino (39/59, 66.10%). A total of 11 (18.33%) users identified as part of the LGBTQ community. Among users who completed both the baseline and the 3-month questionnaires, 91.45% (*n*=107) chose to complete the questionnaires in English. They ranged in age from 11 to 18 (mean 13.83, SD 2.17) years old. The majority were female (58/111, 52.25%). The most commonly self-identified race/ethnicity was Hispanic or Latino (78/107, 72.89%). A total of 15 (14.23%) users identified as part of the LGBTQ community. Table 2 includes detailed information on users’ demographic characteristics.

**Table 2:** Demographic characteristics of users at each time point.

		Completed baseline and midline, n (%)	Completed baseline and endline, n (%)	Completed baseline, midline, and endline, n (%)
**Language**				
	Spanish	2 (3.0)	10 (8.5)	1 (3.8)
	English	65 (97.0)	107 (91.5)	25 (96.2)
**Sex**				
	Female	32 (52.5)	58 (52.3)	10 (43.5)
	Male	27 (44.3)	49 (44.1)	12 (52.2)
	Prefer not to state	2 (3.3)	4 (3.6)	1 (4.3)
**Race/Ethnicity**				
	American Indian or Alaska Native	2 (3.4)	1 (0.9)	1 (4.3)
	Black or African American	5 (8.5)	16 (15.0)	1 (4.3)
	White	12 (20.3)	7 (6.5)	5 (21.7)
	Asian	1 (1.7)	4 (3.7)	1 (4.3)
	Native Hawaiian or Other Pacific Islander	0 (0)	1 (0.9)	0 (0)
	Hispanic or Latino	39 (66.1)	78 (72.9)	15 (65.2)
**LGBTQ^a^**				
	Yes	11 (18.3)	15 (14.3)	5 (20.8)

^a^LGBTQ: lesbian, gay, bisexual, transgender, queer, and questioning.

**Figure 2:**
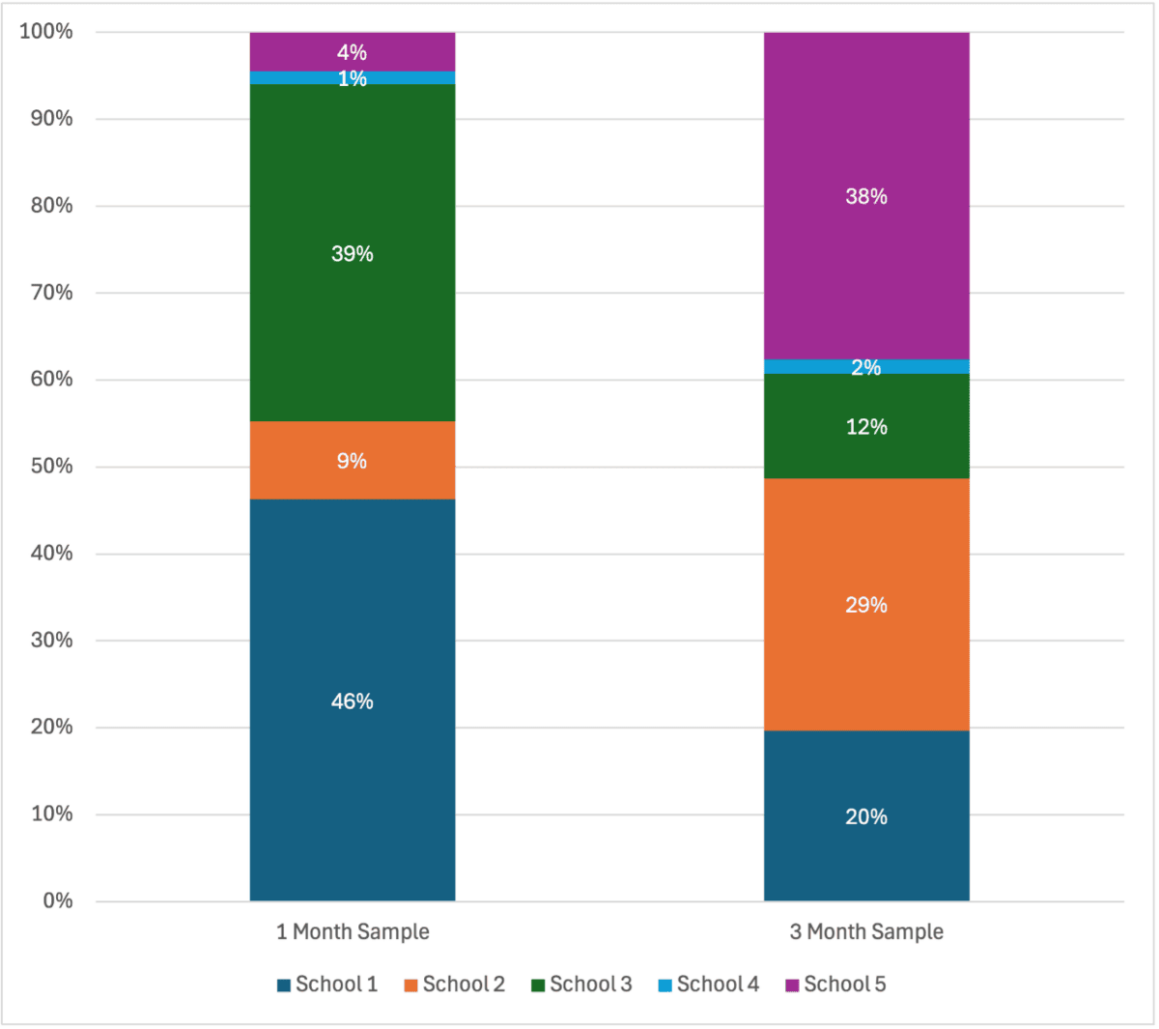
Proportion of the sample that completed 1-month and 3-month surveys from each of the 5 schools.

Results from Wilcoxon and chi-square tests suggested that there were no significant differences in baseline demographic characteristics (age, sex, race/ethnicity, and LGBTQ status) between the users who completed both baseline and 1-month questionnaires and users who completed both baseline and 3-month questionnaires. Additionally, there was no evidence that the 2 groups differed in their baseline scores on the YP-CORE, PHQ-2, GAD-2, BHS-4, or ULS-3. There were 2 key differences between the 2 samples. (1) Users who completed baseline and 1-month assessments had significantly lower expectancies scores at baseline (mean 5.62, SD 2.92) compared with users who completed baseline and 3 month assessments (mean 6.71, SD 2.58, *W*=2572.5, *P*=.02); (2) there were significantly different proportions of students in each school who completed the 1-month versus the 3-month assessments (*χ*^2^_4_=50.64, *P*<.01). Figure 2 displays the different proportions of students from each school that are represented in the 2 samples.

We additionally tested whether baseline YP-CORE scores differed between schools. Baseline YP-CORE scores were significantly different across schools among users who completed both baseline and 1-month questionnaires (*F*_1_=8.41, *P*=.006), but not among users who completed both baseline and 3-month questionnaires (*F*_1_=0.56, *P*=.46). Table 3 portrays baseline YP-CORE scores across schools for both samples.

**Table 3:** Example Young Person’s CORE (YP-CORE) scores at baseline per school, for those who completed the 1- and 3-month surveys.

School	1-month data, mean (SD)	3-month data, mean (SD)
School 1	11.96 (6.59)	14.05 (7.53)
School 2	17.00 (10.39)	13.17 (6.48)
School 3	16.61 (6.16)	17.50 (7.79)
School 4	25.00 (N/A^a^)	11.50 (4.95)
School 5	20.33 (5.86)	16.19 (8.45)

^a^N/A: not applicable.

### Usage Metrics

Table 4 includes data on users’ app use metrics. Results among the sample of users who completed both baseline and 3-month assessments encompass the entire 3-month period (not the period between 1 month and 3 months).

**Table 4:** Usage metrics across time points.

Usage metric	1-month data, mean (SD)	3-month data, mean (SD)
App opens	2.85 (2.85)	4.19 (4.44)
Chat sessions	3.09 (3.15)	3.33 (3.72)
Goals created	0.87 (1.48)	1.14 (1.85)
Journals	0.42 (1.09)	0.29 (0.92)
Videos	0.21 (0.67)	0.40 (1.88)
Mood log	0.12 (0.56)	0.17 (0.65)
Time spent chatting (seconds)	988.5 (1211.66)	1103 (1392.89)
Total activities	4.70 (5.13)	5.33 (6.99)
Chat messages sent	44.45 (44.42)	48.19 (54.63)
Chats completed	1.43 (1.21)	1.49 (1.69)

### Hypothesis 1: Changes in Outcomes Across 3 Months

A paired *t* test showed statistically significant within-person decreases in YP-CORE scores from baseline (*n*=43, mean 14.33, SD 7.43) to 1 month (*n*=43, mean 12.42, SD 7.36; *t*_42_=2.21, *P*=.03) with a small effect size (*r*=0.34). However, a Wilcoxon signed-rank test did not show statistically significant within-person decreases in YP-CORE scores from baseline (*n*=85, mean 14.67, SD 7.75) to 3 months (*n*=85, mean 14.05, SD 8.06; *W*=1821, *P*=.16).

Wilcoxon signed-rank tests did not show statistically significant within-person changes in PHQ-2 scores from baseline (*n*=50, mean 1.42, SD 1.42) to 1 month (*n*=50, mean 1.2, SD 1.55; *W*=264, *P*=.3) or from baseline (*n*=98, mean 1.5, SD 1.61) to 3 months (*n*=98, mean 1.37, SD 1.65; *W*=772, *P*=.29).

Wilcoxon signed-rank tests did not show statistically significant within-person changes in GAD-2 scores from baseline (*n*=53, mean 1.39, SD 1.63) to 1 month (*n*=53, mean 1.17, SD 1.44; *W*=220, *P*=.24) or from baseline (*n*=102, mean 1.69, SD 1.76) to 3 months (*n*=102, mean 1.52, SD 1.87; *W*=1174, *P*=.16).

A Wilcoxon signed-rank test did not show statistically significant within-person changes in BHS-4 scores from baseline (*n*=51, mean 2.98, SD 2.82) to 1 month (*n*=51, mean 3.0, SD 2.79; *W*=278, *P*=.75). However, a Wilcoxon signed-rank test showed statistically significant within-person decreases in BHS-4 scores from baseline (*n*=96, mean 3.62, SD 3.28) to 3 months (*n*=96, mean 2.99, SD 3.21; *W*=1530, *P*=.006) with a moderate effect size (*r*=0.32).

Wilcoxon signed-rank tests did not show statistically significant within-person changes in ULS-3 scores from baseline (*n*=49, mean 4.98, SD 1.76) to 1 month (*n*=49, mean 4.82, SD 1.73; *W*=295, *P*=.56) or from baseline (*n*=102, mean 5.19, SD 1.82) to 3 months (*n*=102, mean 4.88, SD 1.89; *W*=1400, *P*=.06).

Wilcoxon signed-rank tests did not show statistically significant within-person changes in expectancies scores from baseline (*n*=56, mean 5.46, SD 2.94) to 1 month (*n*=56, mean 5.79, SD 3.04; *W*=450, *P*=.6) or from baseline (*n*=103, mean 6.72, SD 2.62) to 3 months (*n*=103, mean 6.15, SD 2.99; *W*=1778, *P*=.10).

### Hypothesis 2: Demographic Predictors of Change in YP-CORE

Results from linear regressions showed there was no evidence that sociodemographic factors (age, race/ethnicity, LGBTQ identity, sex, and school) significantly predicted changes in YP-CORE scores from baseline to 1 month. There was evidence that identifying as part of the LGBTQ community was a significant predictor of lower distress from baseline to 3 months (*t*_57_=–2.07, *P*=.04). There was no evidence that any other sociodemographic factors predicted changes in YP-CORE scores from baseline to 3 months.

### Hypothesis 3: App Use Predictors of Change in YP-CORE

Results from linear regressions showed there was no evidence that total number of activity sessions, videos watched, chat sessions, journal entries created, or goals set predicted changes in YP-CORE scores at 1 month or 3 months.

### Exploratory Analyses

#### Elevated Subsample

In a subsample of users who had clinically elevated distress at baseline (defined as YP-CORE of ≥ 14.1) [25], a Wilcoxon signed-rank test showed statistically significant within-person decreases in YP- CORE scores from baseline (*n*=20, mean 20.6, SD 5.17) to 1 month (*n*=20, mean 16.9, SD 5.97; *W*=128, *P*=.02) with a large effect size (*r*=0.52). Additionally, a Wilcoxon signed rank showed statistically significant within-person decreases in YP-CORE scores from baseline (*n*=42, mean 20.95, SD 5.26) to 3 months (*n*=42, mean 17.71, SD 7.51; *W*=682, *P*=.004), with a moderate effect size (*r*=0.45).

A Wilcoxon signed rank showed statistically significant within-person decreases in GAD-2 scores from baseline (*n*=24, mean 2.38, SD 1.84) to 1 month (*n*=24, mean 1.63, SD 1.69; *W*=122, *P*=.03), however there did not appear to be statistically significant within-person changes in GAD-2 scores from baseline (*n*=44, mean 2.64, SD 1.79) to 3 months (*n*=44, mean 2.11, SD 2.03; *W*=398, *P*=.08).

A paired *t* test did not show statistically significant within-person decreases in BHS-4 scores from baseline (*n*=20, mean 4.9, SD 3.01) to 1 month (*n*=20, mean 4.05, SD 2.96; *t*_19_=1.29, *P*=.21), however a Wilcoxon signed-rank test showed statistically significant within-person decreases in BHS-4 scores from baseline (*n*=40, mean 5.5, SD 3.39) to 3 months (*n*=40, mean 4.3, SD 3.45; *W*=504, *P*=.007) with a moderate effect size (*r*=0.32).

Results on other secondary outcomes (PHQ-2, ULS-3, expectancies) were not significant at 1 month or 3 months.

Results from linear regressions showed that, among users with clinically elevated distress at baseline, identifying as part of the LGBTQ community was a significant predictor of lower distress at 1-month (*t*_6_=–3.04, *P*=.02) and at 3 months (*t*_22_=–2.44, *P*=.02). There was no evidence that any other sociodemographic factors predicted changes in YP-CORE scores at 1 month or 3 months.

There was no evidence that total number of activity sessions, videos watched, chat sessions, journal entries created, or goals set predicted changes in YP-CORE scores at 1 month or 3 months.

#### Differences in Use Between Users Who Did and Did Not See Clinically Significant Decreases in Distress

We categorized users based on whether they showed clinically significant changes in distress. According to Twigg et al [28] the overall reliable change index value for the YP-CORE is 7.9 (including all age bands and genders). Among participants who completed both the baseline and 1-month YP-CORE, 13.95% (*n*=6) saw a clinically significant decrease in distress (scores decreased by 7.9 or more), 2.33% (*n*=1) saw a clinically significant increase in distress (scores increased by 7.9 or more), and 83.72% (*n*=36) did not see clinically significant changes (changes in scores were < 7.9 and > –7.9). Among participants who completed both the baseline and 3-month YP-CORE, 11.76% (*n*=10) saw a clinically significant decrease in distress, 10.59% (*n*=9) saw a clinically significant increase in distress, and 77.65% (*n*=66) did not see clinically significant changes.

At 1 month, users who saw clinically significant decreases in distress opened the app significantly more (*n*=6, mean 5, SD 3.22) compared with individuals who did not see clinically significant changes in distress (*n*=36, mean 2.94, SD 3.14, *W*=163, *P*=.04). Users who saw clinically significant decreases in distress also saved their mood more often (*n*=6, mean 0.83, SD 1.6) compared with individuals who did not see clinically significant changes in distress (*n=*36, mean 0.06, SD 0.33, *W*=141, *P*=.009), spent more seconds chatting (*n*=6, mean 1463, SD 1121) compared with individuals who did not see clinically significant changes in distress (*n*=36, mean 1045, SD 1417, *W*=173, *P*=.02), and sent more chat messages (*n*=6, mean 57.2, SD 24.8) compared with individuals who did not see clinically significant changes in distress (*n*=36, mean 47.8, SD 54.5, *W*=164, *P*=.045). No differences emerged for number of chat sessions, goals created, journal entries, videos watched, or chats completed.

At 3 months, users who had a clinically significant decrease in distress saved their mood significantly more (*n*=10, mean 0.7, SD 1.34) compared with uses who did not have clinically significant changes (*n*=66, mean 0.12, SD 0.57; *F*_2, 82_=3.56, *P*=.03). No differences emerged for number of app opens, chat sessions, goals created, journal entries, videos watched, time spent chatting, total activities, chat messages sent, or chats completed.

#### Differences Between LGBTQ and Non-LGBTQ Users

There was no evidence that LGBTQ users differed in their baseline YP-CORE scores at in either the 1-month (*W*=136.5, *P*=.13) or 3-month samples (*W*=354, *P*=.16). At 1 month, LGBTQ users opened the app significantly more (*n*=11, mean 5.27, SD 4.92) compared with non-LGBTQ users (*n*=49, mean 2.49, SD 2.05; *W*=148, *P*=.02) and spent more time chatting in seconds (*n*=11, mean 1689, SD 1554) compared with non-LGBTQ users (*n*=49, mean 919, SD 1154; *W*=155, *P*=.03). At 1 month, no differences emerged for number of chat sessions, goals created, journal entries, videos watched, mood log entries, chat messages sent, or chats completed. At 3 months, LGBTQ users used the journal tool significantly more (*n*=15, mean 1.2, SD 2.08) compared with non-LGBTQ users (*n*=90, mean 0.18, SD 0.51; *W*=397, *P*<.01) At 3 months, no differences emerged for number of app opens, chat sessions, goals created, videos watched, mood log entries, time spent chatting, chat messages sent, or chats completed.

## Discussion

### Principal Findings

In light of the increased budget and resource limitations for student mental health and wellness in schools, innovative solutions are necessary. This paper reports findings from a pragmatic evaluation of Alongside, a novel digital mental health program designed to provide preventative mental health support and identify students who need more support while remaining cost-effective and scalable. We found evidence that overall distress (our primary outcome) decreased over 1 month among all Alongside users who completed at least one chat outside of the initial onboarding session, although decreases were not sustained 3 months post registration. Due to the timing of the 3-month follow-up correlating with end of the year exams, further controlled studies are necessary to disentangle potential timing effects. In a subsample of users who had elevated distress at baseline, however, findings were both stronger and sustained over time. Findings on secondary outcomes related to depression, anxiety, loneliness, and treatment expectancies were largely null, although some positive effects were seen in hopelessness among the full sample and both anxiety and hopelessness among the elevated subsample. While preliminary evidence suggests some positive results, it is critical that we contextualize them alongside null findings and consider areas for future work.

There are multiple reasons why decreases in overall distress may have been seen among the full sample at 1 month but not sustained at 3 months. First, the Alongside program may be most effective in the short-term for in-the-moment distress, but not currently effective for implementing long-term change. This does not negate the meaningfulness of short-term improvements. In school settings where resources are spread thin, it may prove better to provide easily accessible tools that promote short-term improvements, as opposed to no support at all for those who have sub-clinical levels of distress (ie, those not in need of immediate intervention), which is the common approach currently. Second, a decrease in use of the Alongside program between 1 month and 3 months could explain why users did not see sustained benefits. When comparing app use between the sample that completed the 3-month questionnaires and the sample that completed the 1-month questionnaires, we see marginal differences; for example, the average number of app opens at 3 months (across the entire 3-month period) was 4.19, while the average number of app opens at 1 month was 2.85, a difference of only 1.34. We discuss the unclear relationship between app use and symptom change in more detail later in this discussion, but it is possible that a lack of benefit beyond 1 month could be attributed to low intervention uptake beyond 1 month. Last, there could be discrepancies between the sample of users who completed 1-month questionnaires and users who completed 3-month questionnaires. We found that the proportion of students from each school significantly differed between the 2 samples, and among the users who completed the 1-month questionnaires, distress at baseline significantly differed between users from different schools. At the same time, there was no evidence that baseline distress or demographic characteristics significantly differed between users who completed 1-month questionnaires and users who completed 3-month questionnaires. Still, it is possible that the practical limitations and implementation challenges that Alongside faced in recruiting users to complete questionnaires, discussed in more detail below, contributed to discrepancies between findings at 1 month and 3 months.

Contrary to our hypotheses, we did not see effects on any secondary outcomes related to depression, anxiety, loneliness, or treatment expectancies among the full sample. We caution against interpreting null results in this initial evaluation as a sign to abandon the Alongside program, particularly in light of other potential benefits on short-term distress or benefits among the subsample of users with elevated distress; rather, null findings may reflect that decreases in a largely subclinical sample may not be detectable with the study design. Our findings pointing to stronger effects among the clinically elevated subsample are unsurprising in light of previous research demonstrating larger effect sizes for school-based digital interventions among clinically elevated subsamples of students [30,31], even though Alongside was not specifically developed for this population. At the same time, the results from this evaluation are preliminary and must be interpreted with caution.

Our findings related to Hypothesis 2 suggest that LGBTQ users may see greater decreases in distress than non-LGBTQ users. Otherwise, there were no demographic predictors of changes in overall distress. LGBTQ users may have been more likely to benefit from using the Alongside program because LGBTQ youth often face unique barriers to accessing other mental health care [32]. We also found that LGBTQ users differed from non-LGBTQ users by using several app features more often than non-LGBTQ users. It is possible that the Alongside program is particularly well-suited for dissemination among LGBTQ students. However, these findings are exploratory due to the small sample size of LGBTQ students; replication is necessary to draw strong conclusions. It is also important to note that gender and sexual identity, can be fluid, particularly during adolescence; therefore, these subgroup findings reflect current self-identity. In future research, longitudinal studies can track both intervention effects and identity development over time.

Our findings related to hypothesis 3 suggest that there is no a relationship between users' degree of app use and users' change in overall distress. It is possible that one use may be enough for someone to benefit from, while users who repeatedly come back to a program may do so because they are not getting what they need. Individual differences may better explain the complicated relationship between app use and symptom change. At the same time, we found that users with clinically significant changes in distress used several app features more often than users who did not see clinically significant changes. It is possible that the relationship is not proportional (eg, greater use confers greater impact) but that there is a minimal level of use that can confer some impact, and the degree of impact is individually variable; additional testing is necessary. Alternatively, the strength of the relationship between use metrics and change in distress may exist but may be small enough that our limited sample size was not powered to detect it. In sum, we cannot discount that the degree of program use influences the degree of clinical impact to some extent, but it does not appear to be a particularly meaningful contributor.

Multiple limitations impact our ability to confidently interpret the findings of the current evaluation. First, this was not a randomized evaluation. Without a control group to compare to, we cannot confirm that any positive outcomes were due solely to use of the Alongside program. Simultaneously, we cannot determine whether there were preventative effects such that users who did not see decreases in distress may have otherwise seen increases in distress had they not had access to the program. Second, the primary and secondary outcomes were deficit-focused and did not include well-being measures. The dual-factor model of mental health [33] suggests that well-being and distress are distinct constructs; null findings related to distress do not preclude positive impacts on well-being, however, the current evaluation cannot elucidate the impact of the Alongside program on well-being. Last, Alongside faced a plethora of barriers to implementation that impacted the number of users who responded to questionnaires. One school had over 36% of students screen positive for thoughts of self-harm which contributed to hesitancy around pushing for follow-up assessments as the number of students identified overwhelmed the counseling staff. Additionally, the 3-month endline overlapped with state testing in all Texas schools, leading to difficulty promoting follow-up. Finally, one school asked students to return school devices before the 3-month endline and the survey was not available on personal devices. Only 26 users completed questionnaires at all 3 timepoints, therefore we could not reliably analyze change over time, including all timepoints. The timing of the study in the school year may also have contributed to low numbers of students who responded to questionnaires.

The current evaluation is strengthened by its pragmatic nature and preregistered analysis. The research-to-practice gap is maintained due to a lack of research on real-world implementation, making findings from highly controlled research studies ungeneralizable to the constantly changing and complicated settings in which the intervention is intended to be deployed. By testing an intervention while it is actively being implemented in a nonresearch setting, it is more likely that findings will be reflective of the ultimate impact an intervention can have. Additionally, this analysis was preregistered, and all deviations from the preregistration are reported. Preregistration addresses the “file drawer” problem within the scientific community, wherein only statistically significant results are shared, and null findings are not published. Preregistration is particularly important when considering the lack of transparency that many commercial digital mental health tools maintain. Digital mental companies largely do not test their products through rigorous research at early stages of development. Transparent testing upfront is necessary for companies to then transparently improve their platforms.

This analysis highlights several opportunities for continued research. This study reports initial, small-scale findings, and replication is required. Additionally, randomized evaluations with large numbers of participants are necessary to reliably parse the effects of the Alongside program. Further evaluations may test future iterations of the Alongside program as it is updated to improve its features or add content that may directly influence secondary outcomes such as depression or anxiety. Additionally, hybrid effectiveness-implementation trials may evaluate the Alongside program as implemented via tier 2 for students who are experiencing early signs of mental distress.

### Conclusions

Providing digital mental health tools through schools may help youth to access needed health supports and resources. In this nonrandomized pilot pragmatic evaluation of the Alongside digital mental health tool, we assessed the near-term impact of app use on the self-reported mental health outcomes of middle and high school students. We found that Alongside can confer some short-term benefits, but those benefits are not necessarily sustained in a universal sample. Additionally, the nonrandomized design, attrition, and implementation challenges limit causal inferences and generalizability. Future work may use a larger sample, randomized design, or longitudinal design to further understand when and for whom Alongside may be most effective. While this study takes a valuable step toward understanding digital mental health tools in schools, further validation is necessary.

## Data Availability

The datasets analyzed during this study are not publicly available due to the sensitive nature of the data and per request of the schools participating in the study. Data requests may be submitted to the corresponding author and may be granted pending school partner approval.

## Abbreviations

AI: artificial intelligence
BHS-4: Beck Hopelessness Scale-4
GAD-2: Generalized Anxiety Disorder Questionnaire-2
LGBTQ: lesbian, gay, bisexual, transgender, queer, and questioning
PHQ-2: Patient Health Questionnaire-2
ULS-3: University of California, Los Angeles Loneliness Scale-3
YP-CORE: Young Person’s CORE
